# Political Distinction of Medical Men

**Published:** 1848-09

**Authors:** 


*Political Distinction of Medical Men.*—About thirty members of the National Assembly of France are physicians: of these M. Buchez, a distinguished writer, was the first President, M. Recurt, Vice President, and M. Trélat, Minister of Public Works.

In Prussia, ten physicians have been elected to the National Assembly recently organized; and in Italy, the Pope has appointed the celebrated Italian physician Farni, Under Secratary of State for the papal territories. —*Medical Examiner.*

